# The prosurvival protein BAG3: a new participant in vascular homeostasis

**DOI:** 10.1038/cddis.2016.321

**Published:** 2016-10-20

**Authors:** Albino Carrizzo, Antonio Damato, Mariateresa Ambrosio, Antonia Falco, Alessandra Rosati, Mario Capunzo, Michele Madonna, Maria C Turco, James L Januzzi, Vincenzo De Laurenzi, Carmine Vecchione

**Affiliations:** 1 IRCCS Neuromed, Pozzilli (IS), Italy; 2 Vascular Pathophysiology Unit, Biouniversa s.r.l., c/o University of Salerno, Fisciano (SA), Italy; 3 Department of Pharmacy, University of Salerno, Fisciano (SA), Italy; 4 Department of Medicine and Surgery, University of Salerno, Baronissi (SA), Italy; 5 Cardiology Division, Massachusetts General Hospital and Harvard Medical School, Boston, MA, USA; 6 Dipartimento di Scienze Mediche, Orali e Biotecnologiche, CeSI, Universita' 'G. D'Annunzio' di Chieti e Pescara, Pescara, Italy

## Abstract

Bcl2-associated athanogene 3 (BAG3), is constitutively expressed in a few normal cell types, including myocytes, peripheral nerves and in the brain, and is also expressed in certain tumors. To date, the main studies about the role of BAG3 are focused on its pro-survival effect in tumors through various mechanisms that vary according to cellular type. Recently, elevated concentrations of a soluble form of BAG3 were described in patients affected by advanced stage of heart failure (HF), identifying BAG3 as a potentially useful biomarker in monitoring HF progression. Despite the finding of high levels of BAG3 in the sera of HF patients, there are no data on its possible role on the modulation of vascular tone and blood pressure levels. The aim of this study was to investigate the possible hemodynamic effects of BAG3 performing both *in vitro* and *in vivo* experiments. Through vascular reactivity studies, we demonstrate that BAG3 is capable of evoking dose-dependent vasorelaxation. Of note, BAG3 exerts its vasorelaxant effect on resistance vessels, typically involved in the blood pressure regulation. Our data further show that the molecular mechanism through which BAG3 exerts this effect is the activation of the PI3K/Akt signalling pathway leading to nitric oxide release by endothelial cells. Finally, we show that *in vivo* BAG3 administration is capable of regulating blood pressure and that this is dependent on eNOS regulation since this ability is lost in eNOS KO animals.

The Bcl-2-associated athanogene 3 (BAG3) protein belongs to the family of co-chaperones that interact with the ATPase domain of the heat shock protein HSP70 through a structural domain known as BAG domain (amino acids 110-124).^1, 2^ In addition to the BAG domain, BAG3 contains a WW domain and a proline-rich repeat (PXXP) that can mediate binding to other proteins. Furthermore, two conserved IPV (Ile-Pro-Val) motifs are located between the WW and the PXXP regions and mediate BAG3 binding to HspB8, a member of the HspB family of molecular chaperones.^3^ Therefore, BAG3, due to the adaptor nature of its multi-domain structure, can interact with different proteins.

The *Bag3* gene is constitutively expressed in a few normal cell types, including myocytes, several primary tumors and tumor cell lines, in the peripheral nervous system,^4, 5, 6, 7^ and in the brain. Upregulation of *BAG3* gene expression has been described followed cerebral infarction.^8^ Moreover, BAG3 can be induced in many other cell types by a variety of stressors, mainly through the activation of heat shock transcription factor 1, which in turn is responsible for the transcriptional induction of a number of stress-response genes, including *BAG3*.^2, 9^ Much evidence exists to suggest BAG3 has a role in sustaining cell survival,^2^ through mechanisms that vary depending on the cell context, but in general depend on the ability of BAG3 to modulate levels or localization of apoptosis-regulating proteins, such as IκB kinase gamma (IKKγ),^10^ Bax^11^ or BRAF,^12^ in either an Hsp70-dependent- or independent manner.

While prior data had focused on cellular-based BAG3 biology, we recently described an extracellular form of BAG3 released by stressed cardiomyocytes.^13^ Further study revealed serum BAG3 protein values were increased in patients affected by a more advanced stage of heart failure (HF), suggesting BAG3 protein might be a potentially useful biomarker in monitoring HF progression.^14^ Finally, we demonstrated that BAG3 was prognostic in patients presenting with acutely decompensated HF (ADHF).^15^ Despite these findings, mechanistic explanations for why BAG3 would be elevated in patients with cardiovascular disease are lacking. It is well known that HF is associated with hemodynamic changes, such as reduction of blood pressure levels and increased systemic vascular resistance, depending in a large part on cardiac pump dysfunction.^16^ In the present report, we explored whether BAG3 has nitric oxide (NO) vasorelaxant effects; it was our hypothesis that the protein would regulate vascular tone and mediate blood pressure.

## Results

### BAG3 induces vasorelaxation through the activation of the PI3K/Akt/eNOS signalling

**Figure 1:**
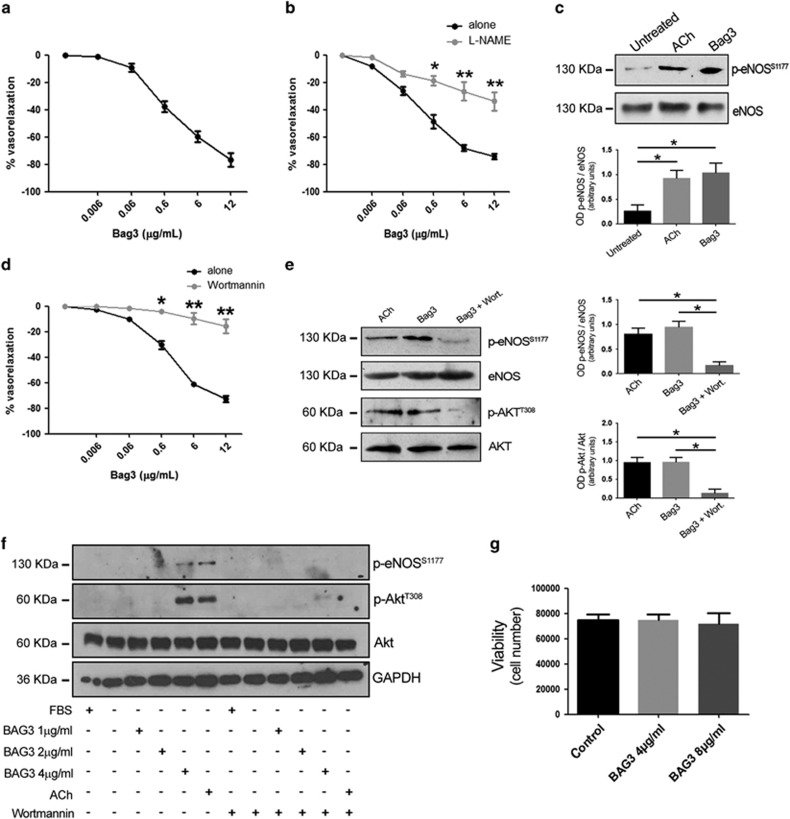
(**a**) Vascular response of phenylephrine-precontracted mice mesenteric arteries to increasing doses of BAG3 (0.006–12 *μ*g/ml) (*n*=6), (**b**) and after 30 min pretreatment with L-NAME (300 *μ*mol/L; *n*=6), *P<0.05, **P<0.01 *versus* BAG3 alone. (**c**) Representative immunoblot of mesenteric arteries, untreated, treated with acetylcholine (Ach) or with BAG3; *right*, Columns are the mean±S.D. of 5 independent experiments. *P<0.05. (**d**) Vascular response of phenylephrine-precontracted mice mesenteric arteries to increasing doses of BAG3 (0.006–12 *μ*g/ml) in presence of PI3K inhibitor (wortmannin 10 *μ*M, 1 h, (*n*=6), *P<0.05, **P<0.01 *versus*. BAG3 alone. (**e**) Representative immunoblot of mesenteric arteries, treated with acetylcholine (Ach), with BAG3 or with BAG3 plus wortmannin; Bottom, columns are the mean±S.D. of 5 independent experiments. (**f**) Representative immunoblot performed on HUVEC treated with different doses of BAG3, Acetylcholine (ACh) in presence or absence of wortmannin. (**g**) HUVEC viability at 24 h after treatment with different doses of BAG3 was assessed by the trypan blue exclusion test. The data presented are the mean±S.D. of three independent experiments

To investigate whether BAG3 could be involved in the modulation of vascular tone, we administered, *ex vivo*, increasing doses of full length recombinant BAG3 in mice mesenteric artery, a typical vascular district involved in blood pressure homeostasis. Our data demonstrate that BAG3 evokes dose-dependent vasorelaxation (Figure 1a), which is significantly blunted by the administration of eNOS inhibitor, N^G^-nitro-L-arginine methyl ester (L-NAME) (Figure 1b). These data suggest the involvement of NO signalling in BAG3 vascular action. Thus, we explored the effect of BAG3 on eNOS phosphorylation on serine^1177^, an activation site of the enzyme.^17^ As shown in Figure 1c, treatment of mesenteric arteries with BAG3 results in eNOS phosphorylation on serine^1177^ to levels comparable to those obtained when treating vessels with acetylcholine, a well-established stimulus for eNOS (Figure 1c).

The PI3K/Akt signalling cascade represents one of the main pathways known to modulate eNOS activation^18^ and PI3K has been shown to mediate BAG3 response in other experimental systems, we therefore tested the possibility that this signalling kinase was also involved in vascular response to BAG3.^19^ Indeed, the administration of wortmannin, a specific PI3K inhibitor, in our experimental setting, significantly reduced BAG3-induced vasorelaxation, thus indicating the involvement of PI3K in BAG3 vascular action (Figure 1d). In addition, further molecular analyses shows that BAG3 treatment induces Akt phosphorylation in threonine^308^. Again, wotmannin blocks both Akt and eNOS phosphorylation induced by BAG3 (Figure 1e).

Taken together, these data suggest BAG3 exerts a vasorelaxing effect through the activation of the PI3K/Akt/eNOS signalling pathway. The involvement of NO signalling in BAG3 vascular action candidate the endothelial layer as the main target of the protein. To clarify this issue, we tested the effect of BAG3 on isolated human endothelial cells. In agreement with the data obtained in vessels, analysis performed on isolated endothelial cells showed that BAG3 induced both eNOS and Akt phosphorylation (Figure 1f). Consistently, also in this experimental setting, the treatment with wortmannin was able to completely abolish BAG3-dependent eNOS and Akt phosphorylation (Figure 1f). It is of note that BAG3 treatment did not influence endothelial cells survival as measured after 24 h (Figure 1g).

### BAG3 reduces the blood pressure *in vivo* and enhances endothelial function

**Figure 2:**
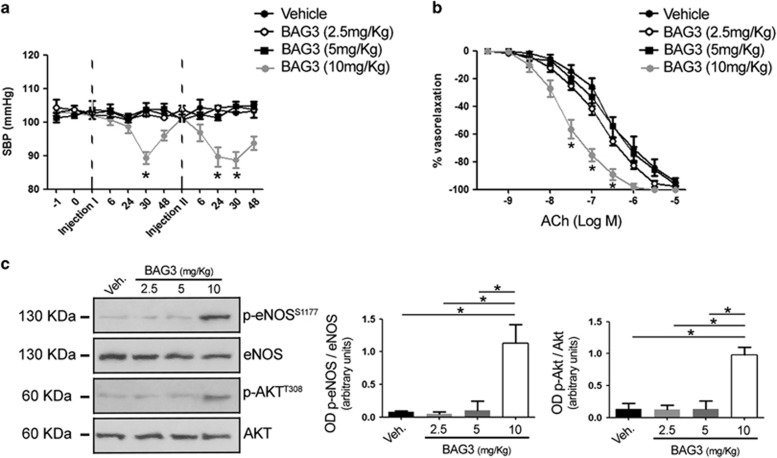
(**a**) Systolic blood pressure (SBP) in C57BL/6 mice treated with vehicle or with different dosage of BAG3 (2.5; 5–10 mg/kg) (*n*=6/group). Dotted line indicate the single intraperitoneal injection of BAG3 or vehicle. The data are given as mean±S.E.M. *P<0.05 *versus* all (unpaired *t*-test). (**b**) Graphs show the dose–response curves of *ex vivo* mesenteric arteries, excised after blood pressure measurement from mice after single intraperitoneal injection with vehicle or BAG3 at different dosage, to acetylcholine (ACh, from 10^−9^ M to 10^−5 ^M). Values are means±S.E.M. *n*=6 experiments. *P<0.05 *versus* all. (**c**) Representative immunoblot for eNOS phosphorylated on Serine^1177^, total eNOS, AKT phosphorylated on threonine^308^ and total AKT in mesenteric arteries obtained from mice treated with vehicle or with different osage of BAG3 (2.5; 5-10 mg/kg); Columns are the mean±S.D. of five independent experiments. *P<0.05

Our results, *in vitro,* prompted us to investigate an *in vivo* potential effect of BAG3. We therefore tested the effect of the intraperitoneal injection of increasing doses of BAG3 (from 2.5 to 10 mg/kg) on blood pressure homeostasis (Figure 2a). In doing so, we observed that the administration of BAG3 at the dose of 10 mg/kg significantly reduced blood pressure levels in contrast to that observed with lower doses. The haemodynamic action of BAG3 was detected after 30 h from the injection, returning to basal levels after 48 h. The administration of a second dose evoked a similar effect on blood pressure homeostasis (Figure 2a) starting at 24 h after the injection. The effect on blood pressure levels was associated with an enhancement of endothelial vasorelaxation and eNOS phosphorylation (Figures 2b and c). In contrast, no effect of BAG3 on vascular function was observed at lower doses.

**Figure 3:**
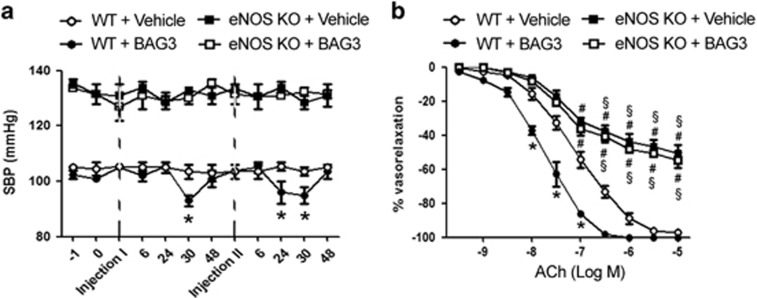
(**a**) Systolic blood pressure (SBP) in C57BL/6 (WT) and in eNOS KO mice treated with vehicle or with BAG3 (10 mg/kg) (*n*=6/group). Dotted line indicate the single intraperitoneal injection of BAG3 or vehicle. The data are given as mean±S.E.M. *P<0.05 *versus* WT+Vehicle. (**b**) Graphs show the dose–response curves of *ex vivo* mesenteric arteries, excised after blood pressure measurement from WT and eNOS KO mice after single intraperitoneal injection with Vehicle or BAG3, to acetylcholine (ACh, from 10^−9 ^M to 10^−5 ^M). Values are means±S.E.M. *n*=6 experiments. *P<0.05 *versus* WT+Vehicle; ^#^*P*<0.05 *versus* WT+BAG3; ^§^*P*<0.05 *versus* WT+Vehicle and *versus* WT+BAG3

To definitively demonstrate the involvement of NO, in the haemodynamic effect evoked by BAG3, we performed experiments in eNOS knockout mice. Our data show that the administration of BAG3 failed to evoke a reduction of blood pressure in eNOS-deficient mice, both at first and second dose, as compared with wild-type mice (Figure 3a). As expected, eNOS KO mice showed endothelial dysfunction as compared with their relative controls. Interestingly, BAG3 administration did not influence endothelial vasorelaxation in eNOS knockout in contrast to that observed in wild-type mice (Figure 3b). These data clearly support a NO-mediated vascular effect of BAG3 *in vivo*.

## Discussion

We have shown that BAG3 has a role in vascular tone regulation and is capable of evoking dose-dependent vasorelaxation. Of note, BAG3 exerts its vasorelaxant effect on resistance vessels, which are the main vascular district involved in the blood pressure regulation. Our data further show that the molecular mechanism through which BAG3 exerts this effect is the activation of the PI3K/Akt signalling pathway, leading to NO release by endothelial cells. More importantly, we show that *in vivo* BAG3 is capable of regulating mice blood pressure and that this is dependent on eNOS regulation since this ability is lost in eNOS KO animals.

To date, the main studies about the role of BAG3 are focused on its involvement in many primary tumors and tumors cell lines. In particular, previous reports have shown that BAG3 is overexpressed in various epithelial cancers, mainly adenocarcinomas, and contributes to cell motility or invasion.^11, 19, 20^ Despite this fact, BAG3 also has a clear role in the cardiac and vascular system. Previous studies have established that BAG3 is constitutively expressed in the heart and skeletal muscle^21, 22^ during cardiomyoblast differentiation, with a role to sustain myogenin expression, suggesting an involvement of BAG3 in late heart development.^5^ Moreover, in cardiomyocytes, BAG3 has been shown to localize at the Z-disc and interact with the actin capping protein, CapZ*β*1, stabilizing myofibril structure and possibly preserving myofibrillar integrity during mechanical stress.^21^ Moreover, we have described a novel signaling pathway in cardiomyocytes that leads to BAG3 upregulation upon exposure to epinephrine through an ERK-dependent upregulation of miR-371a-5p.^23^ Of relevance, the g2252c mutation in the 3'-UTR of BAG3 could potentially result in the loss of the ability to upregulate this protein in response to exposure to high levels of epinephrine, which may be of relevance in diagnoses such as Tako-Tsubo syndrome.^23^

More recently, we have identified an extracellular form of BAG3 released by stressed cardiomyocytes.^13^ Our studies performed in a cohort of patients with chronic HF have revealed increased levels of BAG3 in the serum of patients affected by a more advanced stage of HF, suggesting that BAG3 protein may represent a useful biomarker in monitoring HF progression.^14, 24^ In addition, other reports have proposed elevated serum levels of BAG3 as a novel prognostic marker in patients with acutely decompensated HF (ADHF).^15^ To further support this hypothesis, higher levels of BAG3 have been identified in 20 patients with New York Heart Association (NYHA) class IV symptoms as compared with healthy controls or less symptomatic patients with HF. In this group, elevated concentrations of BAG3 were associated with impending death or need for advanced HF therapies.^14^ However, whether or not the release of BAG3 was just an epiphenomenon or could have a role in the pathogenesis of HF is still unknown.

It is well known that HF is characterized by the inability of systemic perfusion to meet the body's metabolic demands and is usually caused by cardiac pump dysfunction, which could be worsened by persistent hypotension.^25, 26^ This latter phenomenon is characterized by an extensive vasorelaxation of resistance vessels that lead to a critical reduction of arterial blood pressure. Whether abnormalities in BAG3 are participant in the pathophysiology of HF remains unclear, but the vascular role identified in the present report may help to support such a role. Whether exogenous administration of BAG3 as a HF therapeutic would improve clinical outcomes deserves consideration for study.

Our data help to understand the cellular biology and molecular regulation of BAG3 expression in order to design new therapies for the treatment of patients with cardiovascular diseases.

## Materials and Methods

### Experimental animals

All experiments involving animals were conformed to the Guide for the Care and Use of Laboratory Animals published by the US National Institutes of Health (NIH Publication No. 85-23, revised 2011) and were approved by IRCCS INM Neuromed review board. Wild-type C57BL/6 mice or eNOS knockout mice (weighing ~25 g) (Jackson Laboratories, Bar Harbor, ME, USA) have been used to perform blood pressure measurement and for vascular reactivity and molecular studies.

### Vascular reactivity studies

Second-order branches of the mesenteric arterial tree were removed from mice to perform vascular studies. Vessels were placed in a pressure myograph system filled with Krebs solution. First, an analysis of vascular reactivity curves were performed. In particular, vasoconstriction was assessed with 80 mmol/l of KCl or with increasing doses of phenylephrine (from 10^−9^ M to 10^−6^ M) or U46619 (10^−11^ M to 10^−6^ M), in control conditions. Endothelium-dependent and -independent relaxations were assessed by measuring the dilatory responses of mesenteric arteries to cumulative concentrations of acetylcholine (from 10^−9^ M to 10^−5^ M) or nitroglycerine (from 10^−9^ M to 10^−5^ M) respectively, in vessels precontracted with phenylephrine at the dose necessary to obtain a similar level of precontraction in each ring (80% of initial KCl-evoked contraction). Caution was taken to avoid endothelial damage; functional integrity was reflected by the response to acetylcholine (from 10^−9^ M to 10^−6^ M). Vascular responses were then tested administering increasing doses of BAG3 (0.006–12 *μ*g/ml). Full-length recombinant (r) BAG3 was produced by Areta international S.r.l (Varese, Italy). Some mesenteric arteries mounted on a pressure myograph were pretreated with N^G^-nitro-L-arginine methyl ester (L-NAME, 300 *μ*M, 30 min), NOS inhibitor or with PI3K inhibitor (Wormannin, 10 *μ*M, 1 h) before dose–response curve to BAG3.

### Cell cultures

Commercially available human umbilical veins endothelial cells (HUVECs) were purchased from Lonza (Walkersville, MD, USA) and grown in EBM-2 basal medium. Cells were used within the five passages and at 70% confluence for the following sets of experiments. The cells were treated with BAG3 (1,2 or 4 *μ*g/ml) in presence or absence of wortmannin (10 *μ*M, 1 h). Acetylcholine (100 *μ*M, 15 min) has been used as control for the stimulation of eNOS phosphorylation. At the end of treatment, cells have been used to perform immunoblotting analyses. Trypan blue exclusion test was performed to assess cells viability at 24 h.

### Immunoblotting

Pooled vessels or cells were solubilized in lysis buffer containing: 20 mmol/l Tris-HCl, 150 mmol/l NaCl, 20 mmol/l NaF, 2 mmol/l sodium orthovanadate, 1% Nonidet, 100 *μ*g/ml leupeptin, 100 *μ*g/ml aprotinin, and 1 mmol/l phenylmethylsulfonyl fluoride. Then, samples were left on ice for 30 min and centrifuged at 10 621 × *g* for 20 min, and the supernatants were used to perform immunoblot analysis. Total protein levels were determined using the Bradford method. 30 *μ*g of proteins were resolved on 10% SDS-PAGE, transferred to a nitrocellulose membrane and immunoblotted with anti-peNOS^S1177^ (1:1000, Abcam, Cambridge, UK) or with anti-eNOS (1:1000, Abcam), anti-pAKT^T308^, anti-AKT (SantaCruz, Santa Cruz, CA, USA) or anti-GAPDH (Abcam). HRP-conjugated secondary antibodies were used at 1:3000 dilution (Bio-Rad Laboratories, Richmond, CA, USA). Protein bands were detected by ECL Prime (Amersham Biosciences, Uppsala, Sweden) and densitometry analysis was performed using Quantity One software (Bio-Rad Laboratories).

### Blood pressure measurement

C57BL/6 and eNOS deficient mice, 8 weeks old, selected to give a range of blood pressure levels before and after treatment with BAG3 (2.5; 5 or 10 mg/kg), were used for indirect blood pressure measurements using the BP-2000 instruments (Visitech systems, Apex, NC, USA). The tail cuff method was carried out as previously described.^27^

Blood pressure was evaluated in WT, eNOS KO mice under basal conditions and after single oral administration of BAG3 (2, 5; 5 or 10 mg/kg). Animals used as controls were treated in a similar way, but with BAG3 substituted with the vehicle alone. At the end of blood pressure measurements, some vessels were excised to perform vascular reactivity or molecular analyses.

### Statistical analysis

For vascular reactivity studies, statistical analysis was performed by two-way ANOVA followed by Bonferroni *post hoc* test. Immunoblot experiments were presented as mean values and S.D. Group comparisons were made by paired *t*-test. Differences were– considered to be statistically significant at *P*<0.05.

## Abbreviations

BAG3: Bcl2-associated athanogene 3
HF: heart failure
PI3K: Phosphatidylinositol-4,5-bisphosphate 3-kinase
Akt: Protein kinase B
NO: nitric oxide
eNOS: endothelial nitric oxide
PXXP: proline-rich repeat
IPV: Ile-Pro-Val motifs
HSF1: heat shock transcription factor
IKKγ: IκB kinase gamma
Bax: Bcl-2-associated X protein
BRAF: B-Raf proto-oncogene, serine/threonine kinase
Hsp: Heat Shock Protein
ADHF: acutely decompensated heart failure
L-NAME: N^G^-nitro-L-arginine methyl ester
ERK: extracellular signal–regulated kinases
